# Reduced triacylglycerols and lipid droplets are associated with resilience to Alzheimer's disease

**DOI:** 10.1002/alz.71083

**Published:** 2026-02-03

**Authors:** Daan van der Vliet, Luuk E. de Vries, Xinyu Di, Dennis Wever, Brechtje de Jong, Marc T. C. van der Meij, Marielle van der Peet, Amy C. Harms, Thomas Hankemeier, Dick F. Swaab, Inge Huitinga, Joost Verhaagen, Mario van der Stelt

**Affiliations:** ^1^ Department of Molecular Physiology Leiden University & Oncode Institute Leiden The Netherlands; ^2^ Department of Neuroimmunology Netherlands Institute for Neuroscience Institute of the Royal Netherlands Academy of Arts and Sciences Amsterdam The Netherlands; ^3^ Department of Neuroregeneration Netherlands Institute for Neuroscience Royal Netherlands Academy of Arts and Sciences Amsterdam The Netherlands; ^4^ Metabolomics and Analytics Centre Leiden Academic Centre for Drug Research Leiden University Leiden The Netherlands; ^5^ Department of Neuropsychiatric Disorders Netherlands Institute for Neuroscience Institute of the Royal Netherlands Academy of Arts and Sciences Amsterdam The Netherlands; ^6^ Center for Neuroscience Swammerdam Institute for Life Sciences Faculty of Science University of Amsterdam Amsterdam The Netherlands

**Keywords:** Alzheimer's disease, lipid droplets, lipid metabolism, neuroinflammation, resilience

## Abstract

**INTRODUCTION:**

Although it has become clear that alterations in lipid metabolism are associated with Alzheimer's disease (AD), it is unclear how they contribute to both cognitive decline and the pathophysiology of AD.

**METHODS:**

Lipidomics and activity‐based protein profiling (ABPP) were performed in the frontal cortex of control, AD and resilient donors, that is, individuals with AD pathology without cognitive decline.

**RESULTS:**

The most pronounced alterations in lipids were in ω6‐derived oxylipins, which were particularly increased in AD. Triacylglycerols (TAGs) and lipid droplets (LDs) were more abundant in the AD donors compared to the resilient donors. Multi‐omics factor analysis (MOFA) showed that increased ω6‐derived oxylipins and the loss of inhibitory neurons were associated with amyloid beta (Aβ) plaque load.

**DISCUSSION:**

Our multi‐omics data show a molecular response associated with Aβ load shared among AD and resilient donors, but reduced LDs in resilient donors compared to AD.

**Highlights:**

- Comprehensive lipidomics analysis of frontal cortex from controls, Alzheimer's disease (AD) patients and resilient individuals.
- ω6 Oxylipins, markers of neuroinflammation, are increased in both AD and resilience.
- Resilient donors have reduced triacylglycerols and lipid droplets compared to AD.
- Multi‐omics integration shows a molecular response to amyloid beta plaques associated with ω6‐derived oxylipins and loss of interneurons.

## 1 BACKGROUND

Alzheimer's disease (AD) is the most common form of dementia and severely affects memory and other cognitive functions. Effective therapies for the prevention of cognitive decline are currently lacking, and thus there is an urgent need for new therapeutic targets for the development of novel therapies. Recently, the U.S. Food and Drug Administration (FDA) approved several monoclonal antibodies targeting amyloid beta (Aβ) plaques. However, the clinical efficacy of these antibodies is limited and they cause serious side‐effects, such as edema and frequent hemorrhages.^1^ Thus, it is likely that removing Aβ in AD patients alone is not sufficient to halt cognitive decline. This is supported by the fact that some individuals are cognitively intact despite having considerable amounts of AD neuropathology, a phenomenon also known as resilience.^2^, ^3^, ^4^, ^5^ It is thought that these resilient individuals have a different cellular response to pathology, as opposed to AD patients, in which these cellular responses may initiate a cascade leading to cognitive decline. Studies further investigating the possible responses to AD pathology in resilient individuals and AD patients are of potential therapeutic interest.

Recently, our understanding of the cellular and molecular responses to AD pathology in both AD patients and resilient individuals has improved by studying alterations in gene expression and their proteome. Numerous studies have demonstrated a shift toward more reactive glial cells as a reaction to AD pathology,^6^, ^7^, ^8^, ^9^ synaptic vulnerability to oligomeric phosphorylated tau (p‐tau) and Aβ species and mitochondrial dysfunction. Of interest, such changes are often absent or less pronounced in resilient individuals, which could at least partly explain why these individuals remain cognitively intact.^10^, ^11^, ^12^, ^13^ Although these changes are apparent at the gene expression or protein levels, changes in other biomolecules, such as lipids, are not well studied.

Changes in lipid metabolism have been implicated in many processes related to aging and AD. Lipids play crucial roles in neuronal function, energy regulation, myelination, and neuroinflammation, processes that are often altered in AD.^14^, ^15^ Of interest, large‐scale genome‐wide association studies (GWASs) in AD patients have identified multiple risk loci related to cholesterol and lipid metabolism, including apolipoprotein E (*APOE*).^14^ More recently, lipid droplet (LD)–accumulating microglia surrounding plaques have been described in animal models of AD and in AD patients,^16^, ^17^, ^18^ which were associated with increased levels of neuroinflammation.^17^

Besides playing roles in structural membrane formation and energy demands, lipids are also increasingly recognized as signaling molecules. Lipids like endocannabinoids (eCBs), lysophosphatidic acids, prostaglandins, and sphingosine‐1‐phosphate, contribute to synaptic plasticity, memory formation, oligodendrogenesis, bouton formation, and excitability.^19^, ^20^, ^21^, ^22^, ^23^, ^24^ Furthermore, lipids are involved in neuroinflammatory responses through arachidonic acid (AA)–derived neuroinflammatory prostaglandins and other oxylipins.^22^ Various steps in the cascade for AA production and subsequent oxidation to oxylipins have previously been shown to be altered in AD and AD mouse models.^21^, ^25^, ^26^, ^27^, ^28^ However, it remains unclear if lipid signaling contributes to or is altered by the pathophysiology of AD, and how this relates to cognition.

Here we have applied lipidomics and activity‐based protein profiling (ABPP), a methodology to identify active lipid metabolizing enzymes, on gray matter tissue from the frontal cortex of a group of well‐characterized AD patients, resilient individuals, and matched control donors to investigate alterations in lipid metabolism. Furthermore, we integrated both datasets with a previously published bulk RNA‐sequencing dataset to perform a multi‐omics analysis, linking cell types and synaptic processes to changes in lipids. Finally, we confirmed the findings of the lipidomic and ABPP analyses using specialized fluorescent probes, western blot, and immunohistochemistry.

## 2 METHODS

### 2.1 Donors and tissue

Human brain tissue was obtained from the Netherlands Brain Bank (NBB). Written informed consent for use of brain material and clinical data for research purposes and brain autopsies was obtained by the NBB according to international ethical guidelines. Autopsy procedures were approved by the Medical Ethics Committee of the Amsterdam University Medical Center (AUMC), Amsterdam, The Netherlands. Neuropathological assessments were done according to standardized protocols.^29^ Similar donors were selected as described previously.^30^ In brief, donors were selected based on the amount of AD neuropathology and cognition (Clinical Dementia Rating [CDR] scale or Global Determination Scale [GDS]): demented AD patients (CDR 3, Braak 4–6, and Thal ≥ 3), resilient donors (CDR ≤0.5, Braak 3–5, and Thal ≥3), and age‐matched controls (CDR ≤0.5, Braak 1–2, and Thal ≤2). Cases with signs of neurological or psychiatric diseases other than those associated with AD were excluded. Donors with severe comorbid pathology (e.g., cortical Lewy bodies, TAR DNA‐binding protein 43 (TPD‐43), or hippocampal sclerosis) were excluded. Donors were matched as closely as possible for sex, age, pH, post‐mortem delay (PMD), and apolipoprotein E (APOE) genotype (Table 1).

**TABLE 1:** Donor characteristics.

	Snap‐frozen tissue				FFPE tissue			
	Control	AD	Resilient	*p*‐value	Control	AD	Resilient	*p*‐value
*N*	12	12	12		7	7	8	
Sex	6 M/6F	6 M/6F	6 M/6F	> 0.999	2 M/5F	3 M/4F	4 M/4F	0.558
Age	82.2 ± 10.7	82.2 ± 9.9	88.6 ± 8.4	0.188	80.3 ± 10.4	83.9 ± 10.3	85.5 ± 8.7	0.577
PMD	6.0 ± 1.8	5.2 ± 1.0	6.5 ± 1.6	0.119	6.7 ± 1.8	5.0 ± 0.9	6.5 ± 1.6	0.090
pH	6.6 ± 0.3	6.5 ± 0.2	6.4 ± 0.2	0.061	6.4 ± 1.2	6.5 ± 0.3	6.3 ± 0.2	0.233
Braak (B)	1.3 ± 0.7	5.5 ± 0.8	4.1 ± 0.9	< 0.001	1.1 ± 0.7	5.6 ± 0.8	4.4 ± 0.9	< 0.001
Aβ plaque score (A)	0.7 ± 0.5	2.9 ± 0.3	2.4 ± 0.5	< 0.001	0.7 ± 0.5	2.9 ± 0.4	2.3 ± 0.5	< 0.001
Neuritic plaque score (C)	0.1 ± 0.3	2.8 ± 0.6	1.4 ± 0.8	< 0.001	0.0 ± 0.0	2.7 ± 0.8	1.6 ± 0.7	< 0.001
ApoE4+/–	3+/9–	7+/5–	6+/6–	0.441	2+/5–	4+/3–	5+/3–	0.280

Abbreviations: AD, Alzheimer's disease; ApoE4+/–, presence or absence of an apolipoprotein E ε4 allele; FFPE, formalin fixed parafin embedded; PMD, post‐mortem delay.

**RESEARCH IN CONTEXT**

1. **Systematic review**: The authors reviewed recent literature on lipid metabolism in Alzheimer's disease (AD). Although multiple studies have examined lipids in biofluids and tissues of AD patients, this has not been studied in the context of resilience to AD.
2. **Interpretation**: Using a multi‐omics analysis of lipid metabolism, we demonstrate that there is a general response to amyloid beta (Aβ) plaques that is associated with an increase in ω6‐derived oxylipins, markers of neuroinflammation, and loss of interneurons. Furthermore, we provide the first evidence that subtle differences occur in lipid metabolism between AD and resilient individuals, demonstrating lower levels of triacylglycerols and lipid droplets (LDs) in the resilient donors. Our findings indicate that the recently described LD‐accumulating glial phenotype might be reduced in individuals resilient to AD.
3. **Future directions**: Future work could uncover which glial subpopulations contain LDs and how the presence of LDs influences their phenotype. Lipidomics studies on larger patient cohorts might enable a more in‐depth study of interpatient variability. Finally, our study examined a single timepoint in end‐stage AD. It would be interesting to examine lipid composition at earlier timepoints, or follow this in a temporal manner in model systems.

### 2.2 Lipidomics sample preparation

Snap‐frozen human brain tissue from the superior frontal gyrus (SFG) was cut on a cryostat. For each donor, between 15 and 25 mg of tissue was isolated by sectioning ≈10 sections (50 µm). Gray matter was dissected inside the cryostat using pre‐chilled scalpels, collected in pre‐chilled tubes, and immediately put on dry ice. The tissue was homogenized by mechanical lysis with 0.5 mm glass beads in ice‐cold lysis buffer (20 mM 4‐(2‐hydroxyethyl)‐1‐piperazineethanesulfonic acid (HEPES), 1 mM MgCl_2_, 2 U/mL benzonase). A total of 20 µL of lysis buffer/mg tissue was used. The protein concentration was measured using a Bradford assay (Bio‐Rad), the lysates were diluted in lysis buffer to a concentration of 1 mg/mL, and aliquots of the lysates were snap‐frozen in liquid nitrogen and stored at –80°C.

Purchased or synthesized internal standards (IS) were dissolved in methanol, ethanol, chloroform, or acetonitrile (ACN) at different stock concentrations. These stock solutions were further diluted and mixed to make the standard stock solutions and IS stock solutions. The lipids IS mix contained deuterated version of ceramides, (lyso)phospholipids, diacylglycerols (DAGs), triacylglycerols (TAGs), and cholesterol esters. The signaling IS mix contained deuterated version of oxylipins, endocannabinoids (eCBs), free fatty acids, and bile acids. The signaling calibration standards contained oxylipins, eCBs, free fatty acids, and bile acids.

The aliquots of sample lysates (1 mg protein/mL, 78 µL) were thawed on ice in 1.5 mL Eppendorf tubes. To each sample, 10 µL IS work solution was added. Calibration samples were prepared by spiking 10 µL of each calibration standards into 78 µL of water. Extraction was performed by 100 µL extraction buffer (0.2 mM ammonium formate) and 1000 µL extractant (BuOH:EtOAc, 50:50, v/v). Samples were then mixed in a Next Advance Bullet Blender (5 min, room temperature), followed by centrifugation (16,000 × g, 10 min, 4°C). A total of 900 µL of the organic layer was transferred into clean safelock low‐binding tubes and concentrated in a SpeedVac vacuum concentrator (45°C, Thermo Fisher), followed by adding 60 µL of reconstitution solution (MeOH:ACN, 30:70, v/v) and agitating for 15 min. The reconstituted samples were centrifuged (16,000 × *g*, 10 min, 4°C) and 50 µL were transferred into autosampler vials with inserts. Samples were kept at –80°C until mass spectrometry analysis.

### 2.3 Lipidomics LC‐MS/MS measurements

Samples were randomized and run in one batch on three liquid chromatography coupled to tandem mass spectrometry (LC‐MS/MS) platforms. Each batch included quality control (QC) samples and blank samples. QC samples were prepared by combining lysate from all study samples into a single sample, thus representing the average lipid composition of all samples included in the study. This combined sample was divided into six identical QC samples, which were extracted and measured independently. The QC samples are used to assess data quality and stability over the course of LC/MS runs. Method blanks (proc blanks) are used to check for background signal, they were prepared by extracting the lysis buffer (20 mM HEPES, 1 mM MgCl_2_, 2 U/mL benzonase) and have gone through all the steps of the sample preparation procedure with the reagents only.

The signaling lipids platform covers 196 metabolites, including prostaglandins and other oxylipins, N‐acylethanolamines, monoacylglycerols, and free fatty acids. Bile acids are also included in this platform but were not detected. Reference standards were used for each analyte for peak identification, and 47 deuterated IS were used for the correction of variations from sample preparation and LC‐MS/MS runs. A QTRAP 7500 (AB Sciex, Concord, ON, Canada) was coupled to an Exion LC AD (AB Sciex, Concord, ON, Canada). MS/MS experiments were done with a Turbo V source (AB Sciex, Concord, ON, Canada) operated with an electrospray ionization (ESI) probe. An Acquity UPLC BEH C18 column (Waters) was used to measure the samples. The three‐pump LC system consisted of mobile phase A (MPA, H_2_O with 0.1% acetic acid), mobile phase B (MPB, 90% CH3CN/10% MeOH with 0.1% acetic acid), and mobile phase C (MPC, isopropanol (IPA) with 0.1% acetic acid). The injection volume was 5 µL sample stacked with 10 µL of MPA. The flow rate was 0.7 mL/min, and each run takes 16 min. The gradient started at 20% MPB and 1% MPC. The MPB progressed from 20% to 85% between 0.75 and 14 min, whereas the MPC ascended from 1% to 15% between 11 and 14 min, after which conditions were kept for 0.3 min and then the column was re‐equilibrated at initial conditions until 16 min. An ESI source was used with parameters: interface temperature 600°C, curtain gas 45 psi, CAD gas 9 psi, gas 1 and gas 2 both 65 psi. The mass spectrometer operated in polarity‐switching mode and all analytes were monitored in dynamic multiple reaction monitoring (dMRM) mode. Data were acquired using Sciex OS Software V2.0.0.45330 (AB Sciex).

The phospholipids platform covers 1320 nonpolar lipids targets, based on a previously described method.^31^ The samples were measured using three separate acquisition methods with the same LC‐MS conditions but different MRM transitions. Acquisition Method 1 measurement contains classes of phosphatidylcholine, phosphatidylinositol, phosphatidylserine, phosphatidylglycerol, and bis(monoacylglycerol)phosphates. Acquisition Method 2 measurement contains sphingomyelins, hexosylceramides, lactosylceramides, and phosphatidylethanolamines. Acquisition Method 3 measurement contains TAGs. The identification of metabolites in this platform are based on the dimensions of specific MS/MS transitions and retention time, using HILIC columns, the lipids from the same class elute in a narrow retention time window, whereas different lipid classes elute at different retention times. For each class, one or more deuterated IS were used to check the retention times and variations from sample preparation and LC‐MS/MS runs.

A QTRAP 6500+ (AB Sciex, Concord, ON, Canada) was coupled to an Exion LC AD (AB Sciex, Concord, ON, Canada). MS/MS experiments were done with a Turbo V source (AB Sciex, Concord, ON, Canada) operated with ESI probe. A Phenomenex Luna amino column (100 × 2 mm, 3 µm) was used for separation. The MPA was 1 mM ammonium acetate in chloroform:acetonitrile (1:9), whereas MPB was 1 mM ammonium acetate in acetonitrile:water (1:1). The gradient started at 0% MPB. The MPB progressed from 0% to 50% between 2.1 and 11 min, rom 50% to 70% between 11 and 11.5 min, kept at 70% for 1 min, and then the column was re‐equilibrated at initial conditions from 12.6 to 14 min. The injection volume was 2 µL for all three injections. The column temperature was kept at 35°C. The injector needle was washed with isopropanol:water:dichloromethane (94:5:1, v:v:v) after each injection. Assigned MRM peaks from the acquired data were integrated using SCIEX OS (version 2.1.6) software, and signals were corrected using proper internal standards.

The RP MS/MS‐based lipids platform covers 186 lipids, including ceramides, diglycerides, and cholesterol esters. The identification of metabolites in this platform are based on the dimensions of specific MS/MS transitions and retention times, using reversed‐phase columns, the lipids from the same class elute at retention times that can fit into linear regression models involving carbon number and double‐bond number. For each class, one or more deuterated IS were used to check the retention times and variations from sample preparation and LC‐MS runs.

A QTRAP 7500 (AB Sciex, Concord, ON, Canada) was coupled to an Exion LC AD (AB Sciex, Concord, ON, Canada). MS/MS experiments were done with a Turbo V source (AB Sciex, Concord, ON, Canada) operated with ESI probe. An Acquity UPLC BEH C8 column (Waters) was used to measure the samples. The mobile phase consisted of 2 mM HCOONH_4_, 10 mM formic acid (FA) in water (A), ACN (B), and IPA (C). The gradient was the following: starting conditions 10% B and 10% C; increase of B from 10% to 40% between 1 and 2 min; maintaining B at 40% and C at 10% between 2 and 7 min; increase of C from 10% to 45% between 7 and 8 min; maintaining B at 40% and C at 45% between 8 and 10 min; returning to initial conditions at 10.5 min, and re‐equilibration for 1.5 min. The triple quadrupole mass spectrometer operated in polarity switching mode, and all analytes were monitored in dMRM mode. Data were acquired using Sciex OS Software V2.0.0.45330 (AB Sciex).

Assigned MRM peaks from the acquired data from all platforms were integrated using SCIEX OS (version 2.1.6) software, and signals were corrected using proper IS. Blank effects for each analyte were checked by comparing proc blank samples to QC samples. The threshold for blank effects was 40%. The precision and reproducibility of the analytical process were checked using the relative standard deviations (RSDs) of the QCs. The threshold for QC‐RSD was 30%. Any lipid with a blank >40% and RSD >30% was removed from the dataset.

### 2.4 Processing of lipidomics data

All lipids that met the criteria of data quality as described were further processed (629 lipid species). Lipid levels are expressed as response ratios (integrated MRM peaks). The data were further normalized by the sum of all lipids and subsequently log_2_ transformed and centered to zero. Missing values were subsequently imputed by sampling randomly from a distribution with a mean corresponding to the lowest value detected for a given lipid, and a standard deviation (SD) of one‐third of that lipid. This is based on the assumption that if a lipid was not detected, it likely fell below the detection threshold, meaning that the concentration is relatively low.

### 2.5 ABPP using chemical proteomics

The chemical proteomics workflow was based on the previously reported procedures.^32^, ^33^ This procedure has been used previously in human tissue samples and validated extensively in our previous studies.^34^, ^35^ Lysates were prepared as described for lipidomics.

Lysates (100 µL, 1 mg/mL protein) were thawed on ice. For negative controls, 1% sodium dodecyl sulfate (SDS) was added and heated to 95°C for 5 min. Probe cocktail (0.5 mM fluorophosphonate (FP)‐biotin and 0.5 mM tetrahydrolipstatin (THL)‐biotin) was added to the samples and negative controls (ratio 1:50, final concentrations 10 µM for both probes), and the samples were incubated for 30 min at 37°C while shaking (800 rpm). The reaction was quenched by chloroform/methanol precipitation. In sequence, 250 µL H_2_O, 465 µL MeOH, 120 µL CHCl_3_, and 105 µL H_2_O were added to all samples in this specific order, and after each addition the samples were vigorously vortexed. The samples were centrifuged (1500 × *g*, 5 min, RT) and the upper aqueous phase was removed. The pellet and the lower chloroform phase were resuspended in MeOH (500 µL), and the pellet was resuspended by sonicating (10% amplitude, 2 × 10 s). The methanol was removed after centrifugation (20,000 × *g*, 5 min, RT) and the pellet redissolved in 250 µL PBS (0.5% SDS, 5 mM dithiothreitol (DTT)). The samples were sonicated (10% amplitude, 2 × 10 s) and kept at 65°C for 15 min while shaking (800 rpm). After cooling down to RT, 15 µL 0.5 M iodoacetamide in water was added and samples were incubated for 30 min in the dark. The reaction was quenched by adding 5 µL DTT (1 mM DTT), followed by vortexing and centrifugation (20,000 × *g*, 2 min).

From this point on, samples were handled in a flow cabinet, and hairnets and gloves were used to prevent sample contamination. A total of 10 µL slurry (suspension of 50% high‐capacity streptavidin beads, Thermo Fisher 20361) and 30 µL slurry (suspension of 50% control agarose beads, Thermo Fisher 26150) were used for each sample. The beads were washed twice in 6 mL PBS (0.5% SDS) by vortexing and centrifuging (3000 × *g*, 2 min), and washed once with 6 mL PBS by vortexing and spinning down (3000 × *g*, 2 min). The beads were resuspended in PBS and divided in 1.5 mL tubes in a volume of 250 µL per sample. To this suspension the 250 µL protein samples were added. The beads were agitated by overhead turning (2 h, RT). Afterwards, the samples were centrifuged (3000 × *g*, 2 min) and the supernatant discarded by pouring out. The beads were washed 4× with PBS with 0.5% SDS, vortexing, centrifuging (3000 × *g*, 2 min), and discarding supernatant. This step was repeated 5× with PBS to remove SDS. The beads were washed in digestion buffer (100 mM Tris pH8, 100 mM NaCl, 1 mM CaCl_2_, and 2% [v/v] ACN), centrifuged (3000 × *g*, 2 min), and supernatant removed by pipetting. The beads were resuspended in 100 µL digestion buffer containing 0.25 µg trypsin (Promega), and protein was digested overnight at 37°C while vigorously shaking (1000 rpm). To stop the digestion, 100 µL 10% formic acid (FA) in waterwas added and centrifuged (3000 × *g*, 2 min). The samples were prepared by stage‐tip column over an Oasis plate (Waters), which was conditioned by washing sequentially with MeOH, 3:2 ACN/H_2_O with 0.5% FA (solution B) and finally with H_2_O with 0.5% FA (solution A). The peptides were loaded onto the stage tips, washed with solution A, and then eluted into clean safelock low‐binding tubes (Eppendorf). The collected samples were dried in a SpeedVac (45°C, 2–3 h) and stored at –80°C until further use.

The peptides were redissolved in 30 µL LC/MS solution (Milli‐Q/ACN/FA in 97:3:0.1 containing 10 fmol/µL yeast enolase [Waters, product no. 186002325; UniProt P00924]), by vortexing and spinning down briefly. Peptides were then analyzed on a Q Exactive LC‐MS/MS (Thermo Fisher). All gradients and solutions were according to previously published procedures.^32^ Raw spectral data were analyzed by MaxQuant software (v2.0.1.0) to obtain label‐free quantification (LFQ).^36^, ^37^ The “proteingroups.txt” output file from MaxQuant was imported in R. Identified proteins were filtered for potential contaminants identified by MaxQuant, and additional criteria for the proteins were that (1) a protein was identified with two independent unique peptides; (2) the ratio of protein raw intensity of native, heat inactivated lysate was at least 2.0 in at least 5 out of 10 QC pairs; (3) the protein was annotated as a serine hydrolase, or has an annotation in Uniprot as “charge‐relay system” or “nucleophile” as a catalytic residue; and (4) the protein had an LFQ value for >60% of the samples in at least one group. Eighty‐two enzymes met these criteria and were further processed. Missing values were not imputed.

### 2.6 Data analysis and statistics

All downstream analyses were performed in R v4.4.1 using Rstudio. Basic processing and data handling were performed using the tidyverse framework (v2.0.0), and magrittr (v2.0.3). Statistics were calculated using rstatix (v0.7.2) and ggpubr (v0.6.0), and all plotting was done using ggplot2 (v3.5.1), ComplexHeatmap (v2.2.0), cowplot (v1.1.3), and ggrepel (v0.9.5).

### 2.7 Principal component analysis

Principal component analysis (PCA) was performed using the “prcomp” function in base R or the PCAtools package (v2.10.0), with scaling set to FALSE and center set to TRUE. The number of principal components (PCs) to analyze was determined using the “getelbow” function, and correlation of PCs to metadata was done with the “eigencorplot” function of PCAtools, using Spearman correlations, with BH multiple testing correction.

### 2.8 Partial least squares–discriminant analysis

Partial least squares–discriminant analysis (PLS‐DA) was performed using the “ropls” (v1.30.0) package in R, using pathology (control vs AD + resilient) as a response and cognition (AD vs control + resilient) as a response. The fully imputed lipidomics dataset was the data input, further settings were predI = 1, and scale = center. The obtained two dimensions were plotted against each other and data points were colored according to group. Loadings were extracted from the PLS‐DA and plotted on their respective axis using the ComplexHeatmap package.^38^

### 2.9 Differentially altered lipids and proteins

To calculate differentially abundant lipids or proteins from ABPP we used Wilcoxon rank‐sum tests without correction for multiple testing. This test was chosen because >50% of lipids showed a non‐normal distribution as judged by Shapiro–Wilk tests.

ROAST was used to calculate enrichment of lipid sets within the Limma framework (v.3.60.4).^39^, ^40^ The linear model was constructed using a simple linear model design y = ∼0 + group, where group corresponds to control, AD, or resilient, and fed into the roast() function, with indexes referring to specific lipid classes; nrot was set to 10^4^ and set.statistic was set to “mean.”

### 2.10 Gel‐based ABPP and western blot

Lysates were prepared as described. A total of 0.5 µL of a 40× concentrated stock of fluorescent probe in dimethylsulfoxide (DMSO) was added to 19.5 µL of lysate. The used probe was LEI‐612‐BDP‐TMR (200 nM final concentration, 30 min, 37°C). This probe was synthesized in‐house according to published procedures.^41^, ^42^ Full characterization of LEI‐612‐BDP‐TMR will be described elsewhere. After incubation, the lysate was denatured through the addition of 7.5 µL 4× Laemmli buffer (final concentrations: 60 mM Tris [pH 6.8], 2% [w/v] SDS, 10% [v/v] glycerol, 1.25% [v/v] β‐mercaptoethanol, 0.01% [v/v] bromophenol blue) and 15 min incubation at RT. Proteins were resolved on an SDS‐polyacrylamide gel eletrophoresis (PAGE) (10% acrylamide, 29:1 acrylamide:bisacrylamide, Bio‐Rad) for 75 min at 180 V. Subsequently, SDS‐PAGE gels were scanned for in‐gel fluorescence on a ChemiDoc MP (Bio‐Rad) on settings Cy2 (emission 532/50, 120 s exposure), Cy3 (emission 600/50, 120 s exposure), or Cy5 (emission 700/50, 20 s exposure).

The SDS‐PAGE gels were subsequently cut into two pieces; the upper (high molecular weight (MW) part) was stained in Coomassie G250 to assess protein loading. The lower MW part was transferred to nitrocellulose membranes (midi, Bio‐Rad) using a Bio‐Rad Turboblot machine (Mixed MW, 7 min, 25 V). Membranes were cut appropriately and washed in 50 mL tubes using 5% BSA in Tris‐buffered saline with 0.1% Tween‐20 (TBST) for 1 h at RT. Subsequently, membranes were incubated using primary antibodies against ABHD6 (Cell Signaling, D3C8N, 1:1000) in 5% BSA in TBST overnight at 4°C. The next day, membrane was washed three times in TBST, and subsequently incubated with the appropriate secondary antibodies coupled to horseradish peroxidase (HRP,mSanta Cruz). Development of the luminescence was performed using a chemiluminescence kit (Bio‐Rad), and scanned on a ChemiDoc MP. The gel and blot images were further analyzed with Image lab software (v6.1, Bio‐Rad). Bands were quantified and the relative intensity was normalized on the Coomassie staining for protein loading normalization.

### 2.11 RNA‐sequencing data preparation

RNA‐sequencing data were obtained as described previously.^30^ Raw counts were loaded into R using an EdgeR‐voom‐Limma pipeline. The counts were loaded into a DGEList object and subsequently normalized using the trimmed‐mean method (TMM) from EdgeR (v 4.2.1). Counts were equally distributed. Genes were filtered for high expression using the cpmByGroup function. Only genes with a counts per million (CPM) corresponding to a minimum of 10 counts in at least one group (control, AD, or resilient) were kept. This delivered 18,158 genes in 35 samples for further processing. Log_2_ CPMs were calculated using EdgeR's cpm() function with log set to TRUE. Subsequently, the log_2_ CPMs were adjusted for sex and batch using the RemoveBatchEffect() function from Limma.

### 2.12 Multi‐omics factor analysis (MOFA)

Multi‐omics factor analysis (MOFA) was performed using the R package MOFA2 (v1.8.0) using a python dependency with Basilisk (v1.10.2). The input data were the full ABPP data (82 enzymes), the full lipidomics data (629 lipids), and a restricted RNA‐seq dataset. The RNA‐seq data were restricted based on variance, with highly variable genes selected by using a cutoff of 1.5 × mean log_2_ SD. In addition, only protein‐coding genes were kept. This yielded 1699 genes for the input of MOFA. The MOFA model was trained with largely default settings. Scale_views was set to TRUE to avoid excessively variant genes overruling more subtle changes in the lipidomics data. Convergence mode was set to “medium” and maximum iterations were 2000.

Derived factors were filtered based on minimum variance explained: we kept only those factors that explained at least 5% variance in at least one data modality. This led to six factors being kept for further analysis. Five of six factors were normally distributed as tested by Shapiro tests (*p*‐values > 0.05). Factors were then tested across groups using two‐sided Student's *t*‐test with Benjamini‐Hochberg multiple testing correction.

Biological pathways associated with the MOFA factors were determined with fgsea (v1.30.0) and clusterProfiler (v4.12.0), using the feature weights to rank the genes or lipids. Genes were related to gene ontology (“biological process”); lipids were grouped into lipid classes as described above. Factors were correlated (Pearson) to cell‐type abundance based on deconvoluted RNA‐seq data and to plaque and p‐tau load from previously published data.^30^

### 2.13 Immunohistochemistry and immunofluorescence

Adjacent tissue used for the lipidomics and ABPP was used to cut 10 µm cryosections on a cryostat and was stored at –80°C until use. For the Oil Red O (ORO) staining, two sections per donor were brought to room temperature and fixed in 4% paraformaldehyde (PFA) in PBS for 15 min. Sections were washed twice in 60% isopropanol for 5 min and then incubated in ORO solution (0.3 g/L in 5:4 IPA:water) for 30 min at 60°C. Sections were rinsed in 60% isopropanol, then water, and finally stained with hematoxylin for 30 s. The slides were coverslipped with Mowiol 4–88 mounting medium. Imaging of the sections was performed with an Axioscan Z1 (ZEISS, Oberkochen, Germany) at 20× magnification. ORO signal was quantified using Qupath (v0.5.0). Per section, two regions of interest (ROIs) were outlined covering all cortical layers of the gray matter. Within the ROI, a signal with twice the optical density of the background was considered ORO signal. The optical density (OD) was multiplied by the surface area of the total ORO signal divided by the total area of the ROI. Within the same ROI, the cell counter function of Qupath was used on the hematoxylin signal, after which positive cells were automatically counted based on the ORO signal.

For the immunofluorescent staining, formalin‐fixed paraffin‐embedded (FFPE) tissue from the frontal cortex of the same donors was used. Briefly, FFPE sections were deparaffinized in xylene and rehydrated graded ethanol series. Antigen retrieval was performed using citrate buffer at pH 6 in a microwave at 800 W for 10 min. The antibodies against Perilipin‐2 (PLIN2, Progen Biotechnik; 1:200), ionized calcium‐binding adapter molecule 1 (Iba1, WAKO; 1:250), glial fibrillary acidic protein (GFAP, Cy3 conjugated, Sigma‐Aldrich, 1:500), and NeuN (Millipore; 1:500) were diluted in diluent buffer (1% bovine serum albumin (BSA), 0.5% triton in TBS) overnight at 4°C. Secondary antibodies (donkey anti‐mouse Cy3, donkey anti‐guinea pig 488 or donkey anti‐rabbit Cy5, Thermo Fisher Scientific; 1:400) were diluted in the diluent buffer and incubated at room temperature for 1 h. Sections were treated with 0.1% Sudan Black B in 70% EtOH for 5 min and nuclei were visualized with DAPI. Sections were coverslipped with Mowiol 4–88. Representative pictures were taken with a Leica TSC SP5 microscope (60× magnification, 1056 × 1056 and 100 Hz).

## 3 RESULTS

**FIGURE 1:**
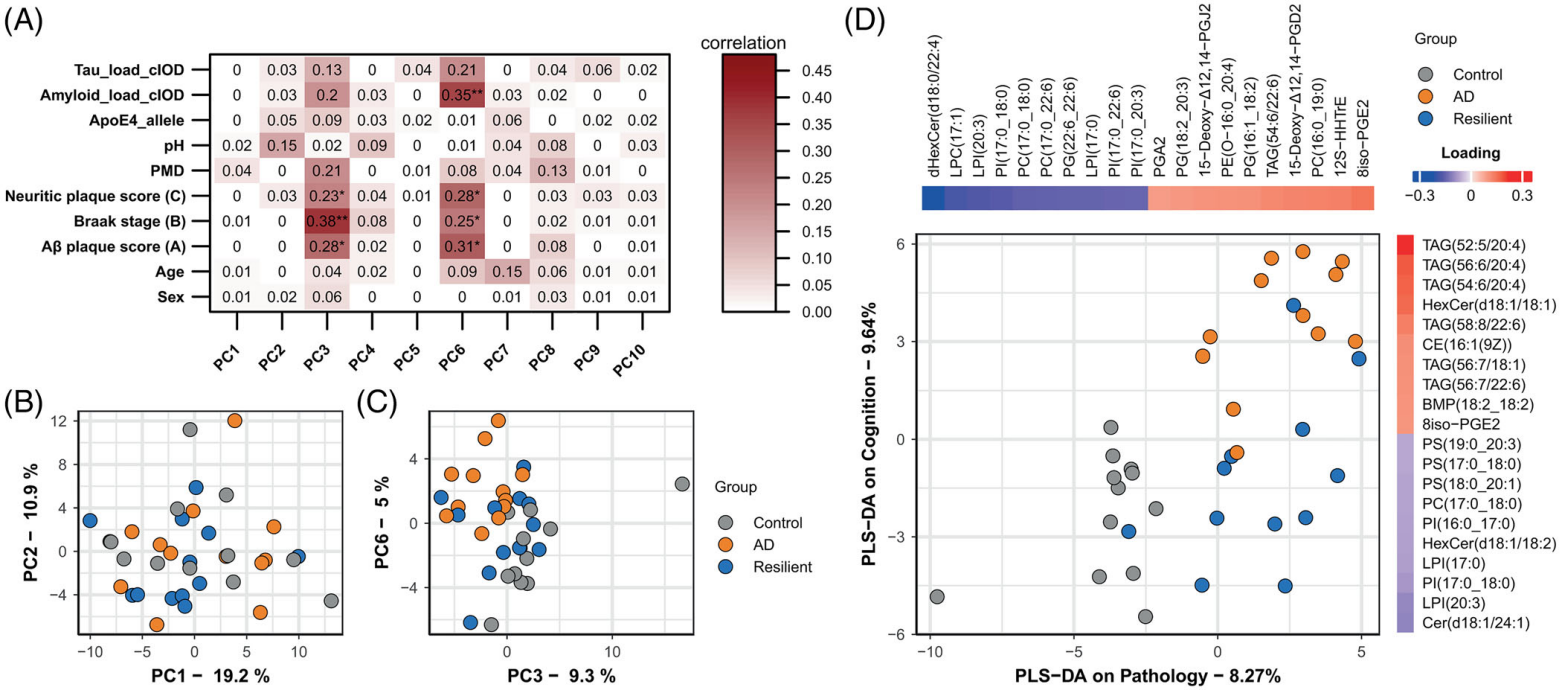
Heterogeneous lipidomic data can be separated by group based on triacylglycerols (TAGs) and oxylipins. (A) Correlations of metadata and the first 10 principal components (PCs). (B) Principal component analysis (PCA) plot of PC1 and PC2 does not separate groups. (C) PCA plot of PC3 and PC6 shows separation between groups. (D) Partial least squares–discriminant analysis (PLS‐DA) shows separation of groups mainly driven by TAGs and oxylipins. The loadings of the top 10 positive and negative loadings are plotted alongside their respective axis. cIOD, corrected integrated optical density; pH, pH of the cerebrospinal fluid (CSF); PMD, post‐mortem delay.

To study alterations in lipid metabolism, we first analyzed the lipid content of the gray matter in the superior frontal gyrus (SFG) from 12 control, AD, and resilient individuals (Table 1), using targeted LC/MS‐MS. We analyzed 629 lipid species across 31 lipid classes, covering a wide range of lipid classes including phospholipids, plasmalogens, sphingomyelins, ceramides, bis(monoacylglycerol)phosphates, acyl‐glycerides, and free fatty acids, and their oxidized derivatives (oxylipins). An unbiased analysis of the main sources of variance using principal component analysis (PCA) showed that most variance in the lipidome did not relate to disease classification (Figure 1A–B). The first two principal components (PCs) also did not relate to donor characteristics like age, post‐mortem delay (PMD), pH of the CSF, or sex, indicating large sources of variability between individuals for currently unknown reasons (Figure 1A). Relating the first 10 PCs to disease characteristics did; however, show that PC3 and PC6 correlated with AD pathology. A plot of these components separated controls from AD, with resilient individuals overlapping with both groups (Figure 1C).

Partial least square‐discriminant analysis (PLS‐DA) was used to identify components that best separated the groups (Figure 1D). As donors were selected based on their amount of pathology and cognition, we used those parameters as input for PLS‐DA. The neuropathological scores were significantly different between AD and resilient individuals versus control donors, whereas there was no difference between AD versus resilient individuals (Table 1). PLS‐DA on AD pathology and cognition revealed that oxylipins were driving the separation between controls and AD and resilient individuals, whereas triacylglycerols (TAGs) contributed to the difference between controls and resilient donors from demented individuals, albeit explaining only a small percentage of the variance.

### 3.1 Inflammatory oxylipins from ω6 free fatty acids are upregulated in response to pathology

**FIGURE 2:**
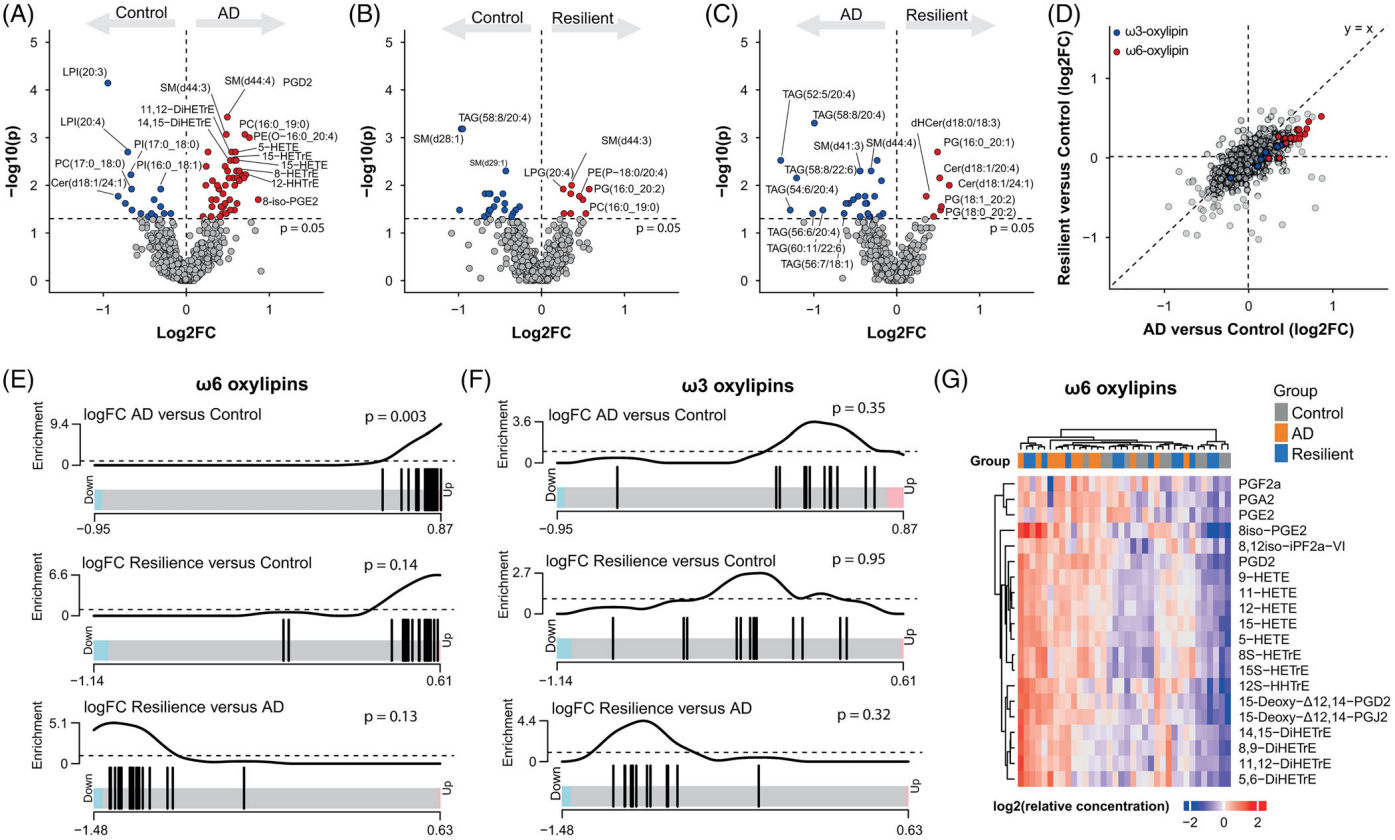
ω6 Oxylipins are increased in Alzheimer's disease (AD) donors. (A–C) Differentially abundant lipids between the three groups in this study. *p*‐values were calculated by Wilcoxon rank‐sum tests. (D) Quadrant plot of fold changes from AD versus control and resilient versus control. ω3 Oxylipins and ω6 Oxylipins are indicated in blue and red, respectively. (E–F) Barcode plots of enrichment analysis using rotation gene‐set testing (ROAST) shows that ω6 oxylipins (E) but not ω3 oxylipins (F) are enriched in AD. (G) Heatmap of normalized ω6 oxylipin levels, presented as z‐scores, shows that these are generally higher in the AD donors.

Next, we analyzed differentially abundant lipids between the groups (Figure 2A–C, Table S1). The fold changes of both AD and resilient groups compared to controls correlated strongly with each other (Figure 2D, Pearson's correlation coefficient R = 0.65, *p* < 2.2 × 10^−^^1^ ^6^), indicating a similar lipidomic response to AD pathology. Oxylipins, products of free fatty acid oxidation and markers of neuroinflammation, showed the most changes between AD patients and controls (Figure 2A). In resilient individuals, oxylipins trended to increase, but this did not reach statistical significance (Figure 2B, D).

Notably, only ω6‐derived oxylipins, not ω3, showed positive fold changes (Figure 2D). The enrichment of ω6‐derived oxylipins was confirmed by rotation gene‐set testing (ROAST) in the AD versus control groups (*p* = 0.003), but not in the resilient individuals versus controls (*p* = 0.14). ω3 Oxylipins were not enriched in any comparison, suggesting that the increase in oxylipins in AD is selective for ω6 free fatty acids (Figure 2F). This may be due to decreased ω3 oxylipin precursors docosahexaenoic acid (DHA, 22:6‐ω3) and eicosapentaenoic acid (EPA, 20:5‐ω3) (Figure S1A,B). No changes were found in AA (20:4‐ω6) and dihomo‐gamma‐linolenic acid (DGLA, 20:3‐ω6) levels, precursors to the ω6 oxylipins (Figure S1C,D). Taken together, these data show that ω6 oxylipins are more abundant in the cortex of AD donors and follow a similar, but less pronounced, trend in resilient donors.

### 3.2 Lower TAG levels and LDs in resilient individuals

**FIGURE 3:**
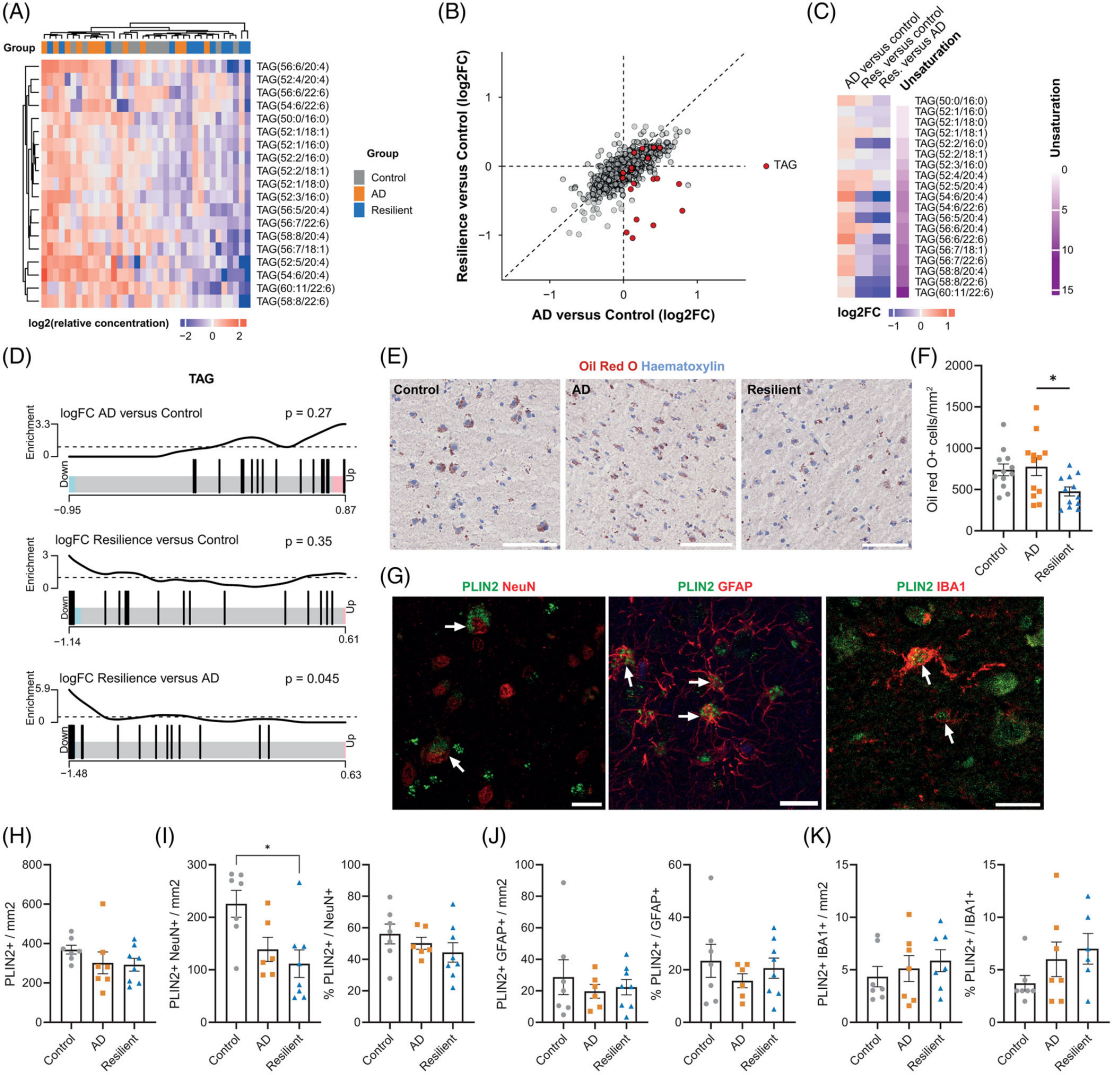
Triacylglycerols (TAGs) and lipid droplets are reduced in resilient donors. (A) Heatmap of normalized TAG levels shows separation of the resilient from Alzheimer's disease (AD) donors. (B) Quadrant plot of fold changes from AD versus control and resilient versus control, showing that some TAGs are less abundant in the resilient group, while more abundant in the AD group. (C) Heatmap of fold changes of the different TAGs showing that mainly the more unsaturated TAGs are less abundant in the resilient group. (D) Enrichment analysis using ROAST shows that TAGs are less abundant in the resilient group. (E) Representative Oil Red O (ORO) of a resilient donor staining in the superior frontal gyrus of the different groups. (F) Quantification of ORO‐positive cells in the cortex across groups. (G) PLIN2, a marker of lipid droplets, co‐localizes with NeuN–, GFAP–, and Iba1‐positive cells. Representative pictures are from the same AD donor. Scale bars in E are 100 µm and in G are 20 µm. (H) Quantification of PLIN2+ cells per mm^2^. (I–K) Cell type–specific quantification of PLIN2+ cells for neurons (NeuN), astrocytes (GFAP), and microglia (Iba1). Data are expressed as the number of double positive cells per mm^2^ for PLIN2 and NeuN (I), GFAP (J), or Iba1 (K), alongside, the percentage of PLIN2+ cells among all cells positive for the cell type marker. Data are presented as mean ± SEM. *p < 0.05, **p < 0.01 from ANOVA with Tukey post hoc test.

Because we observed a reduction for several species of TAGs in resilience compared to AD (Figure 2C), and several TAGs associated with cognition in the PLS‐DA (Figure 1D), we further investigated TAG levels across the groups. We observed separation of AD and resilient donors by hierarchical clustering (Figure 3A). A subset of TAGs showed negative fold changes in the resilient group, while they had positive fold changes in the AD group (Figure 3B). These changes were more pronounced in TAGs with a higher degree of unsaturation, whereas more‐saturated TAGs remained generally unchanged (Figure 3C). Enrichment analysis revealed a significant decrease in TAG levels in resilient donors compared to AD (*p* = 0.045, Figure 3D), suggesting lower total TAG levels in the resilient donors. Because TAGs are major components of lipid droplets (LDs), these data may reflect alterations in LDs in resilient individuals compared to AD patients. Of note, we measured only two species of cholesterol esters (CEs), another major lipid component of LDs, one of which showed an increase in AD but not in resilience (Figure S1E–F).

To obtain an independent line of evidence for alterations in LD content between resilient and AD donors, neutral lipids were visualized in adjacent tissue sections using Oil Red O (ORO) staining. A higher number of cytoplasmic LDs was observed in cortical cells of AD donors compared to resilient donors (Figure 3E–F: *F* = 4.09, *p* = 0.027; AD vs control, *p* = 0.942; resilient vs control, *p* = 0.071; AD vs resilient, *p* = 0.034). No significant change was observed between AD and control donors. We did not find an association of both TAGs and LDs with the presence an *APOE* ε4 allele (Figure S2). Staining of PLIN2, an integral LD membrane protein marker, revealed that astrocytes, microglia, and neurons across all donor groups contained LDs (Figure 3G), suggesting that the changes in LDs are not limited to one specific cell type.

The number of PLIN2 positive cells was; however, not different between groups (Figure 3H: *F* = 1.15, *p* = 0.338), also when stratified on cell types markers for astrocytes (GFAP, Figure 3J: *F* = 0.368, *p* = 0.697) or microglia (IBA1, Figure 3K: *K* = 1.27, *p* = 0.551). Of interest, there was a reduction in the number of PLIN2+ neurons in the resilient compared to the control group (Figure 3I: *F* = 5.64, *p* = 0.013; AD vs control, *p* = 0.080; resilient vs control, *p* = 0.012; AD vs resilient, *p* = 0.740). This effect was driven by the loss of neurons in the resilient cortex, as the percentage of PLIN2 positive neurons was not altered. Due to the scarcity of the material of the used donor selection, not all donors used in the lipidomics and ORO staining were used for the PLIN2 staining, possibly resulting in underpowered results to pick up the differences between the groups. However, the number of ORO‐positive cells did not correlate with the number of PLIN2 positive cells (Figure S2F). This might indicate that analyzing a protein component of LDs does not reflect lipid content.

### 3.3 ABPP uncovers changes in enzyme activities in response to pathology

To investigate which lipid‐metabolizing enzymes were potentially involved in the changes in oxylipins and TAGs, we performed activity‐based protein profiling (ABPP), a technique to enrich active enzymes from native tissues using chemical probes.^43^ Specifically, we used fluorophosphonate and β‐lactone probes to target serine and cysteine hydrolases, which are intricately involved in lipid metabolism in the brain.^44^

**FIGURE 4:**
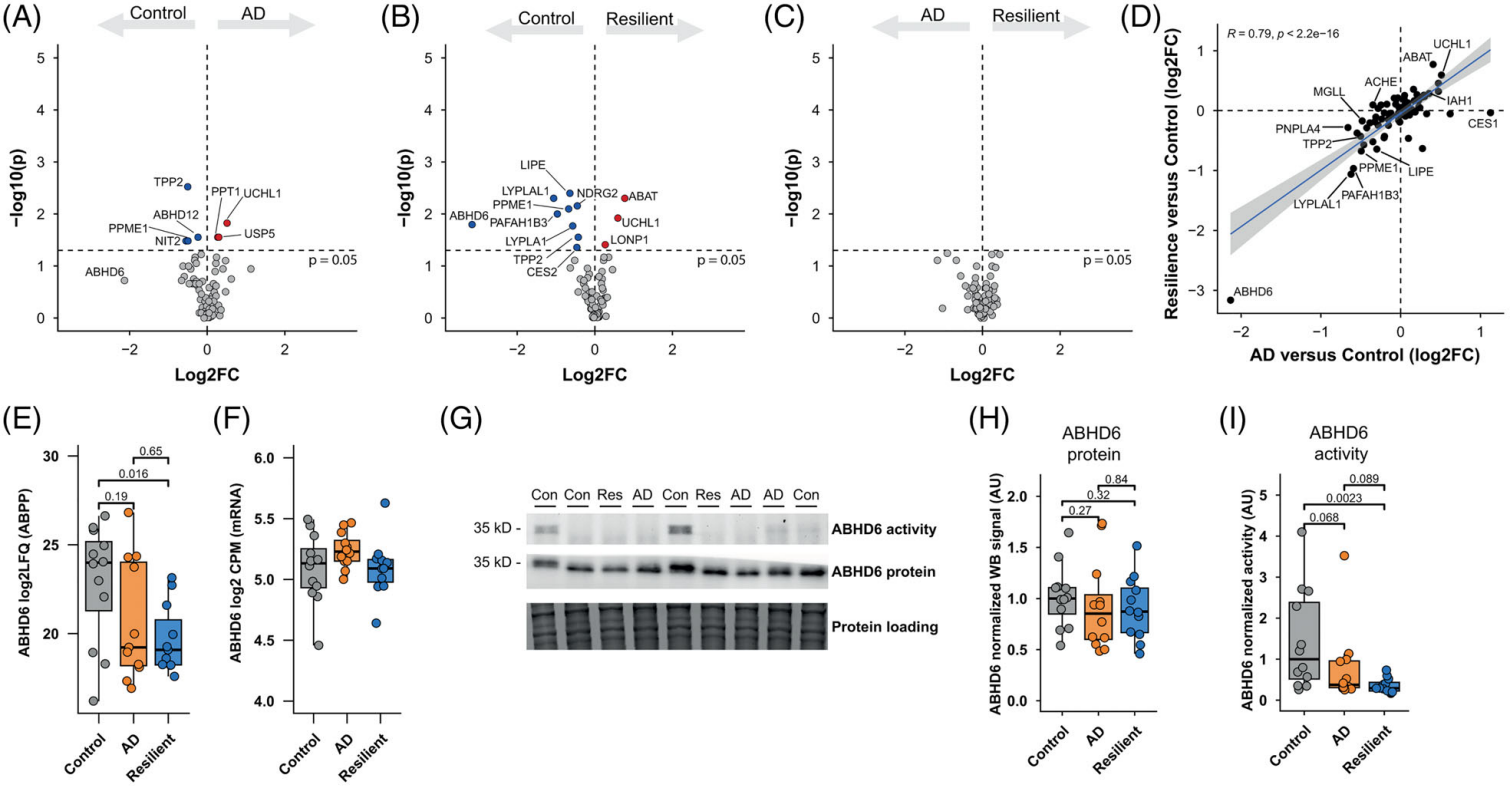
Activity‐based protein profiling shows similar changes in enzyme activities between Alzheimer's disease (AD) and resilient donors. (A–C). Differentially active enzymes between the three groups in this study. *p*‐Values were calculated by Wilcoxon rank‐sum tests. (D) Quadrant plot of fold changes from activity‐based protein profiling (ABPP) results between AD versus control and resilient versus control show that the changes in resilient and AD donors are highly correlated. (E) ABHD6 levels from the ABPP between the groups, showing lower activity levels of ABHD6 in the resilient group. *p*‐Values are the raw *p*‐value from A to C. (F) There are no differences in mRNA levels of *ABHD6*. Transcript levels are derived from a previously generated bulk RNA‐seq dataset.^30^ (G) Representative gels of ABHD6 activity by fluorescent probe LEI‐612‐BDP‐TMR, and ABHD6 protein expression by western blot across the three groups. Full gels can be found in Figure S4. (H) Quantified protein levels of ABHD6 from panel G by western blot. (I) Quantified activity levels from panel G. *p*‐Values represent Wilcoxon rank‐sum tests (*N* = 12 for each group).

We quantified relative enzyme activities for 82 enzymes (Table S2). The activity of these did not correlate with the expression of their respective genes, possibly due to post‐transcriptional and post‐translational regulation (Figure S3). Consistent with the lipidomics dataset, we observed small changes in enzymatic activities across groups. The activity of tripeptidyl peptidase 2 (TPP2) and protein phosphatase methylesterase‐1 (PPME1) were reduced in both AD and resilient donors, whereas ubiquitin C‐terminal hydrolase L1 (UCHL1) was increased in both (Figure 4A, B). Furthermore, alpha/beta‐hydrolase domain containing 6 (ABHD6), 4‐aminobutyrate aminotransferase (ABAT), or platelet‐activating factor acetyl hydrolase 1b catalytic subunit 3 (PAFAH1B3) were altered between control and resilient donors (Figure 4B). Hormone‐sensitive lipase (HSL, gene name *LIPE*), a promiscuous lipase involved in the hydrolysis of TAGs, DAGs, and CEs and thus linked to lipid droplets,^45^ was reduced in resilient individuals and trended to decline in AD (Figure 4B, D).

No changes were observed between AD and resilient donors (Figure 4C). Most fold changes were similar between resilience and AD groups (Figure 4D), indicating that enzyme activities mainly associate with AD pathology.

Notably, ABHD6 was the most downregulated enzyme in the cortex of resilient patients compared to control donors, with a similar trend in AD donors (Figure 4D,E). Remarkably, ABHD6 activity varied by several orders of magnitude across donors, whereas *ABHD6* mRNA expression showed no differences between groups and a much smaller variability^30^ (Figure 4F), suggesting a disconnection between protein activity and gene expression. Western blot analysis confirmed that ABHD6 protein levels were consistent across all samples, whereas gel‐based ABPP analysis revealed that ABHD6 activity was significantly lower in resilient individuals, with a negative trend in AD donors (Figure 4G‐I, Figure S4: resilient vs control, *p* = 0.0023; AD vs control, *p* = 0.068, AD vs resilient, *p* = 0.089). Overall, these results show that decreased ABHD6 activity is associated with AD pathology in a manner that is independent of its expression in both resilient and AD donors.

### 3.4 Multi‐omics data integration couples oxylipin levels to loss of inhibitory neurons in AD and resilience

**FIGURE 5:**
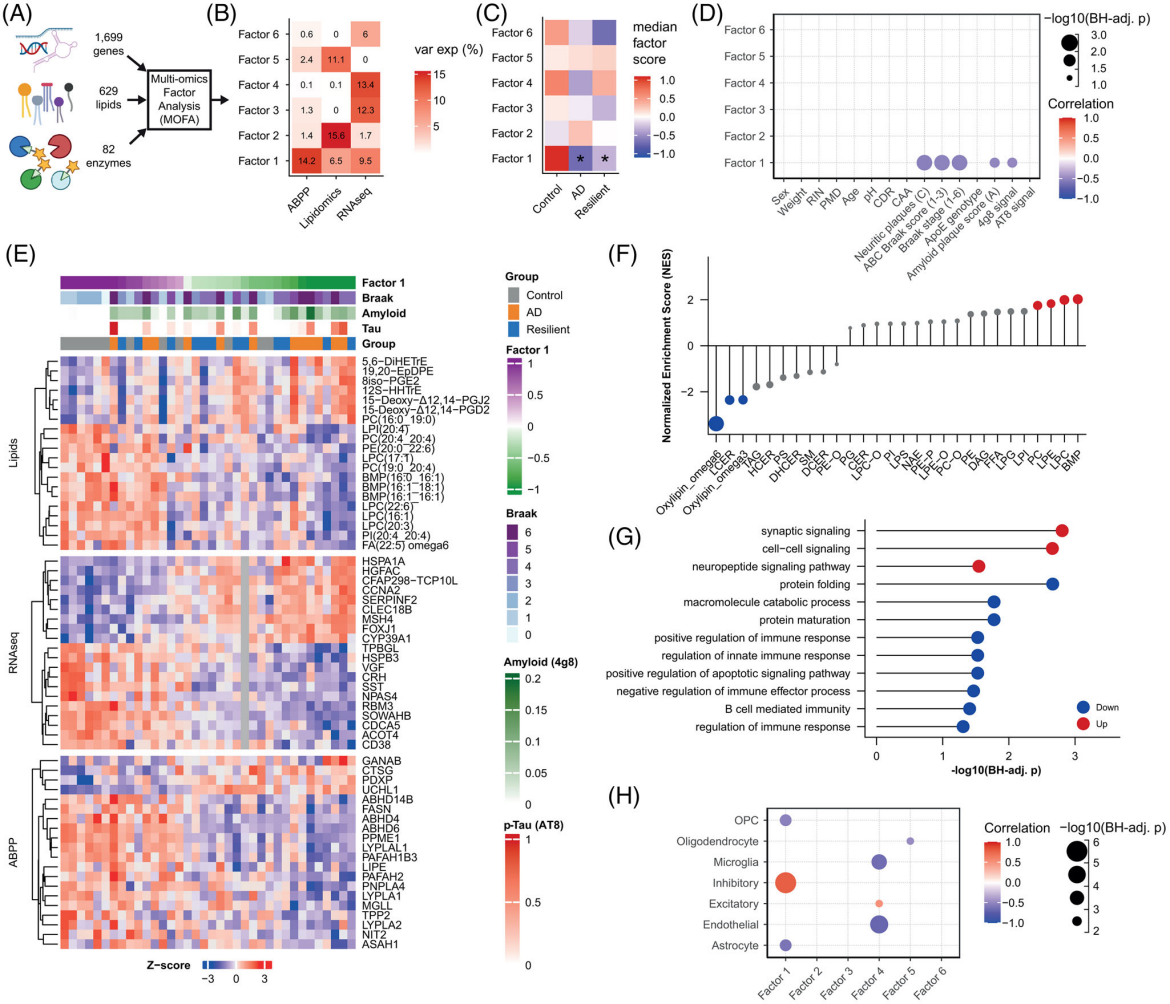
A multi‐omics response in donors with Alzheimer's disease (AD) pathology that is associated with amyloid beta (Aβ) plaque levels. (A) Input data of the different data modalities for the multi‐omics factor analysis (MOFA) analysis. (B) MOFA identified six factors that each explained at least 5% of the variance in at least one data modality. (C) Median factor scores per group, *q < 0.05, Student's *t*‐test with BH correction. (D) Correlation (Pearson) plot of donor characteristics and pathological parameters with the MOFA factors. (E) Heatmap of the top 20 variables associated with Factor 1 from all three data modalities. Data are depicted as *z*‐scores. Legends indicate per sample the group, quantified p‐tau pathology (AT8) and Aβ plaques (4G8), Braak stage, and Factor 1 score. (F) Enrichment analysis of lipid species associated with Factor 1 shows that mainly ω6 oxylipins associate with Factor 1. Data are plotted as normalized enrichment scores (NES). (G) Gene‐set enrichment analysis of genes associated with Factor 1. Data are represented as the BH‐adjusted *p*‐value for enrichment of Gene Ontology (GO) terms positively (red) or negatively (blue) associated with Factor 1. Only GO‐terms with *q* < 0.05 are shown. (H) Correlation of MOFA factors with cell type abundances determined by deconvolution of RNA‐seq data. Data represent Pearsons correlations with BH correction for multiple testing.

Finally, to link the lipid and enzyme activity patterns with the global transcriptome and cell types, we integrated three datasets using multi‐omics factor analysis (MOFA).^46^ We trained the MOFA model on the lipidomics (*N* = 36, 629 lipids) and ABPP (*N* = 36, 82 enzymes) data, together with the most variable genes from a previously generated transcriptome dataset (*N* = 35, 1699 genes)^30^ (Figure 5A; Figure S5). We limited the derived factors to those explaining at least 5% variability in one data modality. Six orthogonal factors were identified, each capturing independent variance across the datasets (Figure 5B; Figure S5).

Only Factor 1 displayed significant association with the three groups, being significantly lower in both AD and resilient donors (Figure 5C). It captured variance from all three data sources (Figure 5B). Factor 1 was significantly correlated with neuropathological scores for AD (Figure 5D), specifically showing a negative correlation with quantified Aβ plaques (by 4G8), but not with p‐tau (AT8). This suggests that Factor 1 reflects a response to Aβ plaques. Factor 1 did not relate to age, PMD, or other disease‐unrelated parameters, showing that it is a neuropathology specific multi‐omics response (Figure 5D).

The genes, lipids, and enzymes with the highest loadings of Factor 1 included oxylipins, heat shock protein‐related genes, *SST* and *VGF*, and monacylglycerol lipase (MGLL) and ABHD6 (Figure 5E; Figure S6). Enrichment analysis using the lipid and gene loadings indicated that ω6 oxylipins were negatively associated with Factor 1, capturing their upregulation in response to Aβ pathology (Figure 5F). In addition, genes related to synaptic signaling were positively associated with Factor 1, whereas genes related to protein folding and immune responses were negatively correlated (Figure 5G; Figure S7). This suggests that Factor 1 reflects a loss of synaptic signaling and increased immune responses and heat shock proteins in the AD and resilient cortex, in relation to Aβ plaque load.

Next, we correlated the MOFA factors to cell type abundances based on cell type deconvolution of the transcriptomics data.^30^ The proportion of astrocytes and oligodendrocyte precursor cells (OPCs) were negatively correlated to Factor 1, possibly indicating gliosis. In contrast, inhibitory neurons showed a strong positive correlation with factor 1 (R = 0.78, *p *= 4.6 × 10^−^^8^; Figure 5H), indicating that increased inflammatory ω6 oxylipins associate with loss of inhibitory neurons in both AD and resilient donors. In conclusion, multi‐omics data integration captured a response to Aβ plaques, characterized by inflammatory ω6 oxylipins, gliosis, a loss of inhibitory neurons, and reduced synaptic signaling.

## 4 DISCUSSION

How alterations in lipid metabolism and signaling might contribute to the pathophysiology of AD is so far not well understood. By using a multi‐omics approach, we provide evidence for alterations in inflammatory ω6 oxylipins, loss of inhibitory neurons, and a decrease in synaptic signaling that are associated with Aβ plaques. Overall, whereas most changes in lipid metabolism were similar in the AD and resilient groups, we found lower levels of TAGs and LDs in the resilient donors specifically.

Increased TAGs and cholesterol esters, major components of lipid droplets, were previously found close to plaques in AD, particularly in glial cells.^16^, ^47^, ^48^ Increased LDs in glial cells have been associated with more proinflammatory states^16^, ^17^ and in animal models, microglia form LDs after Aβ exposure, which is accompanied by a decrease in Aβ phagocytosis.^48^ Green et al. recently reported lipid‐laden microglial states in AD, associated with the development of AD pathology.^8^ Our data could indicate that such microglial states might be reduced in resilient donors. As these lipid‐laden microglial states might be more proinflammatory,^49^ a reduction of proinflammatory microglial subtypes may be expected in resilient donors, which is in line with previous research.^10^, ^50^, ^51^ In addition, a reduced lipid load in microglia was recently described to enhance amyloid plaque clearance in a mouse model of AD.^52^ Although others have shown that LDs are strongly associated with *APOE* ε4^16^, ^47^, ^53^ in our data TAGs and LDs did not correlate with *APOE* ε4. In the current study, TAGs and LDs were found in all cell types, which may explain why in our results there is no correlation with *APOE* ε4. In addition, donors included in our study predominantly had one allele of the *APOE* ε4 variant, whereas previous studies primarily related to biallelic *APOE* ε4. Previous reports described an increase of LDs in AD compared to control donors,^16^, ^48^ a finding not replicated in our study. This is possibly driven by different population characteristics, as differences in the amount of pathology, number of patients with a homozygous *APOE* ε4/ε4 genotype but also differences in studied brain regions could result in different amounts of lipid droplets.

Taken together, the alterations in LDs between the resilient and AD donors may reflect a different glial response to pathology in resilience compared to AD, but further research is warranted to confirm differential abundance of LDs between AD, control, and resilient individuals.

The lipid class with the most significantly altered lipids was the ω6 oxylipins, which were also found in the multi‐omics response and are in line with previous observations.^54^ Oxylipins have been associated with inflammatory processes in AD, in which ω6‐derived oxylipins are generally considered pro‐inflammatory, whereas ω3‐derived oxylipins can reduce neuroinflammation.^55^, ^56^ Another class of closely associated lipids, the specialized pro‐resolving mediators (SPMs) also have important roles in protecting against neuroinflammation. Although they were part of our targeted lipidomics platform, we did not detect these lipids, most likely due to their low concentration. Because mainly ω6‐derived oxylipins were enriched in AD donors and in MOFA Factor 1, this may indicate increased levels of neuroinflammation in these donors. Of interest, mainly unsaturated TAGs were more abundant in AD patients compared to resilient donors, which can serve as precursors to oxylipins. This could suggest that the reduced TAGs in the resilient donors possibly have resulted in lower levels of oxylipins. The ω6 oxylipin response in the cortex of AD donors indeed was stronger than in resilient donors. Furthermore, MOFA Factor 1 correlated with Aβ plaques but not with p‐tau, indicating that processes captured by this factor are possibly a specific response to Aβ plaques. This response may be similar in both AD and resilient donors, suggesting that this could either be a general response or a response associated with early stages of AD pathophysiology, rather than related to diminished cognition, as the resilient donors are cognitively intact. Early features of AD are synaptic loss^57^, ^58^ and loss of inhibitory interneurons,^59^, ^60^, ^61^, ^62^ possibly driven by Aβ‐induced neuroinflammation. Our data indicate that these processes might already be ongoing in the cortex of resilient individuals, despite having intact cognition. These data further illustrate that when focusing on certain mechanisms, resilient individuals can be seen as “preclinical” AD patients that are still able to maintain cognition.^2^

The enzyme activities determined by ABPP did not reveal enzymes that could explain the difference in TAGs and LDs in this cohort. Most enzyme activities changed similarly in both the resilient and AD donors compared to controls, indicating that this group of enzymes might be altered as a general reaction to neuropathology. We further profiled the monoacylglycerol‐hydrolyzing enzyme ABHD6. ABHD6 was significantly reduced in resilient donors and showed a trend toward lower levels in AD. ABHD6 is involved in the hydrolysis of 2‐AG to AA^63^, ^64^ and is reduced in hippocampal neurons in AD patients.^26^ Besides its role in lipid metabolism, ABHD6 is also involved in α‐amino‐3‐hydroxy‐5‐methyl‐4‐isoxazolepropionic acid receptor (AMPAR) trafficking.^65^ Strikingly, ABHD6 activity was uncoupled from its expression levels, which, to our knowledge, has not been reported so far. One possibility might be that specific point mutations in these donors affect ABHD6 activity but not its protein levels. Alternatively, post‐translational regulation could cause a disconnection between protein levels and its activity. In addition, UCHL1 activity levels were higher in resilient donors and showed a trend in the same direction for the AD donors, which has been linked to lower levels of myloid precursor protein (APP) and Aβ.^66^ TPP2 activity was significantly reduced in both resilient donors and AD patients. TPP2 is involved in antigen processing, cholecystokinin degradation, cell growth, and DNA damage repair,^67^, ^68^, ^69^ and lower levels have been linked to cognitive deficits in mice.^70^ Both enzymes showed a similar direction in the AD and resilient donors, indicating that this could be a general reaction to AD pathology. Of interest, others have shown that higher levels of the protein PAFAH1B3 might be associated with resilience.^71^, ^72^ In the ABPP data, PAFAH1B3 was lower in the resilient donors compared to the control donors. It plays an important role in brain development,^73^ and its paralog PAFAH1B2 has been shown to reduce Aβ peptide production.^74^ The difference with the current study may be explained by the difference in measuring protein abundance with proteomics or activity with ABPP.

There was significant disease‐unrelated variability in both the lipidomic and ABPP datasets, highlighting the need for many donors to be included in omics‐studies like the current study. However, the donors used here have previously been comprehensively characterized to minimize confounding factors, such as latent or comorbid disease.^30^ This approach ensured the inclusion of resilient donors and excluded a large number of preclinical AD donors, which are often included by others,^71^ and of which it is uncertain if they would become resilient and postpone the onset of AD or develop dementia sooner if pathology would progress. Despite having 12 donors for each group, our study appeared underpowered to quantify the subtle changes in lipid composition with more statistical certainty. Furthermore, it might be possible that the bulk approaches used in the current study are not sensitive enough to detect local differences. Many of the previously indicated roles of lipids in AD are microglia related, which are less abundant than other cell types. Furthermore, many changes could happen in close vicinity to plaques. Recently, spatial mass spectrometry approaches have shown increased phospholipid species around plaques in AD patients^75^ and differences in gangliosides in Aβ plaques from AD patients compared to Aβ plaques found in controls.^76^ Future studies should focus on whether changes in lipid metabolism in relation to AD are found in specific microglia populations or surrounding specific Aβ plaque types.

In conclusion, we show that there is a general response to Aβ plaques that is associated with an increase in ω6 oxylipins and loss of interneurons. Furthermore, we provide the first evidence that subtle differences occur in lipid metabolism between AD and resilient individuals, demonstrating lower levels of TAGs and LDs in the resilient donors.

## AUTHOR CONTRIBUTIONS

Luuk E. de Vries and Dick F. Swaab performed the donor selection and additional diagnostics. Daan van der Vliet, Luuk E. de Vries, Xinyu Di, Dennis Wever, and Marc T. C. van der Meij collected data. Data collection was supervised by Thomas Hankemeier, Amy C. Harms, and Mario van der Stelt. Daan van der Vliet, Luuk E. de Vries, Dennis Wever, Brechtje de Jong, Marielle van der Peet, and Marc T. C. van der Meij analyzed data. Luuk E. de Vries and Daan van der Vliet drafted and revised the manuscript and prepared the figures with input from all authors. Luuk E. de Vries, Daan van der Vliet, Inge Huitinga, Joost Verhaagen, and Mario van der Stelt designed and coordinated the study. All authors read and approved the final manuscript.

## CONFLICT OF INTEREST STATEMENT

The authors declare no conflicts of interest. Any author disclosures are available in the supporting information.

## ETHICS APPROVAL AND CONSENT TO PARTICIPATE/PUBLICATION

Written informed consent for a brain autopsy and for the use of the brain material and clinical data for research purposes was obtained by the Netherlands Brain Bank according to international ethical guidelines and were approved by the Medical Ethic Committee of the Vrije Universiteit Medical Center, Amsterdam, The Netherlands.

## Supporting information



Supporting information

Supporting information

Supporting information

Supporting information

## Data Availability

No custom code was developed for this study. Mass spectrometry data for activity‐based protein profiling is available through the ProteomeXchange Consortium via the Proteomics Identifications database (PRIDE) with accession code PXD057993. RNA sequencing data used in this study were obtained and previously reported by de Vries et al.^30^ and are available through the Gene Expression Omnibus with accession number GSE261817. Other data and R code are available upon request to the corresponding authors.
